# Tapered Tiles Modulate Flexibility in Segmented Armadillo-Inspired Armor

**DOI:** 10.1093/icb/icaf055

**Published:** 2025-05-27

**Authors:** Julia B Teeple, Karly E Cohen, Theodore Stankowich, E W Misty Paig-Tran, Cassandra M Donatelli

**Affiliations:** Biological Sciences, California State University Fullerton, 800 State College Blvd. Fullerton, CA 92831, USA; Biological Sciences, California State University Fullerton, 800 State College Blvd. Fullerton, CA 92831, USA; Friday Harbor Laboratories, University of Washington, 620 University Way, Friday Harbor, WA 98250, USA; Biological Sciences, California State University Long Beach, 1250 Bellflower Blvd, Long Beach, CA 90840, USA; Biological Sciences, California State University Fullerton, 800 State College Blvd. Fullerton, CA 92831, USA; School of Engineering and Technology, University of Washington Tacoma, 1900 Commerce St, Tacoma, WA 98402, USA

## Abstract

Biological segmented armors integrate mineralized tiles with soft tissues, forming a structure that is both puncture resistant and flexible. In the 9-banded armadillo *Dasypus novemcinctus*, scapular and pelvic buckler osteoderm tiles are hexagonally shaped, tapering from the superficial face down to the deep face. Each osteoderm is embedded in the dermis and adjacent osteoderms are connected to one another via connective Sharpey’s fibers. Our study hierarchically investigated the relationship between armor geometry, connective fibers, and soft supporting layers during flexion. We used micro-CT scans to inform the design of simplified 3D-printed buckler osteoderm models with 3 taper angles, 2 types of connective layers of different compliances (elastic and rigid), and one soft silicone rubber layer. Resistance to bending for 18 model combinations were tested using a 3-point bend test. We found that tapered tiles form a “sweet spot” between flexibility and rigidity. Tapered geometry decreased the stiffness of the system, while models without tapers greatly increased the stiffness via increased tile interactions. The stiff fabric set a limit for bending, regardless of taper type, and there was no additive effect when combining stiff and elastic fabrics. The silicone rubber increased the flexural stiffness of the model and helped to redistribute forces. This study further demonstrates that armadillo armor is complex and relies on hard-soft interfaces to resist bending and to translocate damaging forces. When creating bio-inspired models, it is imperative to take biological complexity into account, yet test the system hierarchically to better predict the role of the geometry as well as the material (hard and soft elements).

## Introduction

Many species have evolved morphological anti-predator mechanisms, including spines, toxic sprays/secretions, and biological armors (Emlen 2008; Stankowich and Campbell 2016; Crofts and Stankowich 2021). Osteoderms, bony deposits in the dermis, represent a form of biological armor widespread across taxa (Vickaryous and Sire 2009; Frýdlová et al. 2023; Ebel et al. 2025). The development of these mineralized structures varies across linages, forming through bone metaplasia (transformation of connective tissue) and/or intramembranous ossification (osteoblast-driven bone formation) (Vickaryous and Sire 2009; Williams et al. 2022; Ebel et al. 2025). While primarily proposed to function as armor, osteoderms have also been proposed to serve other non-protective functions like thermoregulation (Seidel 1979; Clarac et al. 2018), stabilizing movement (Witzmann 2009), morphological displays (Main et al. 2005; Ebel et al. 2025), and mineral reservoirs (Dacke et al. 2015; Curry Rogers et al. 2016).

**Fig. 1:**
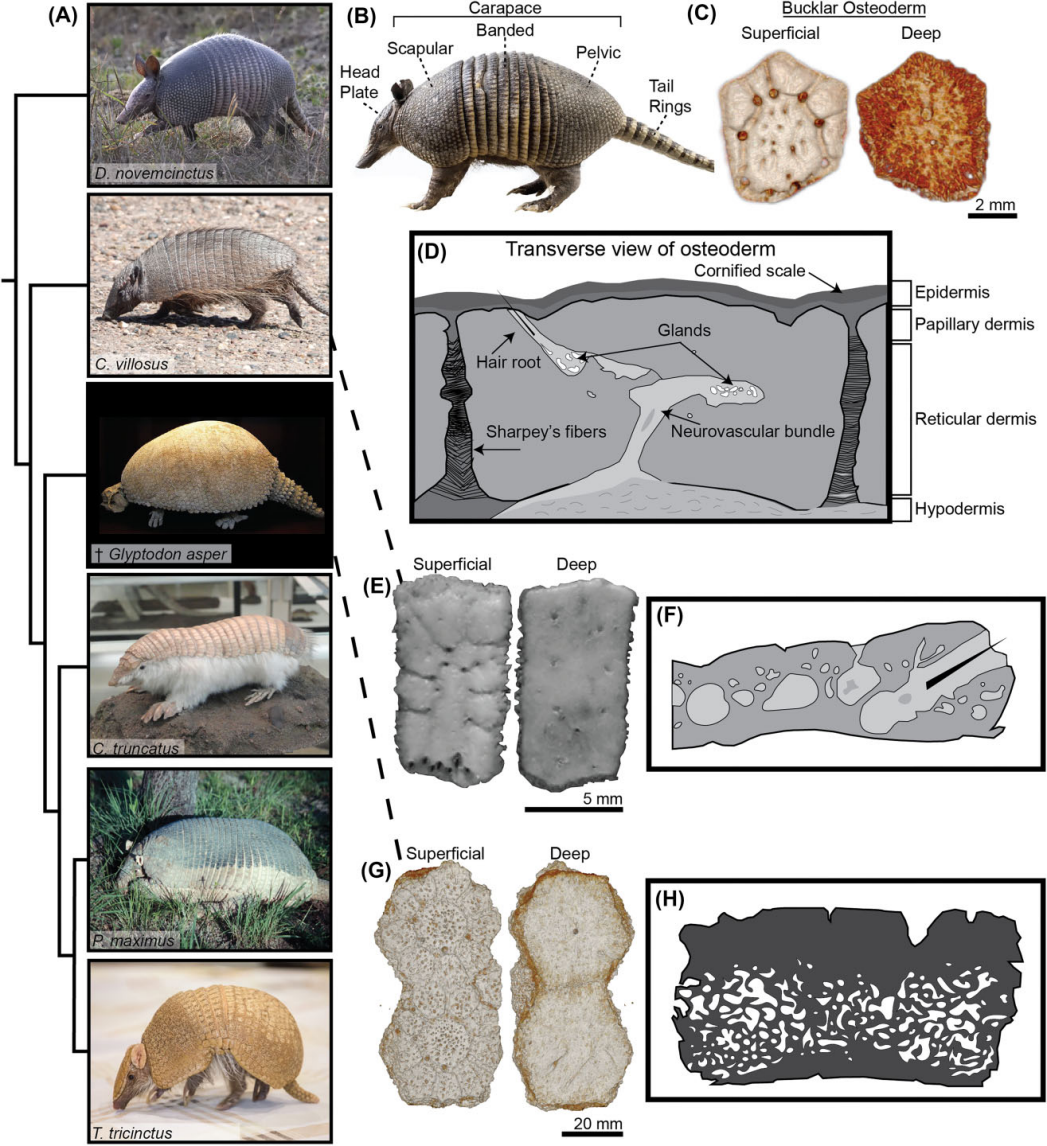
(**A**) Armadillos have a diversity of armor types. (**B**) *D asypus novemcinctus* highlights 2 distinct osteoderm regions (fixed buckler and moveable bands). (**C**) *D asypus novemcinctus* osteoderm micro-CT view of superficial and deep faces, displaying characteristic polygonal shape and nodular patterning. (**D**) Crosssectional view of fixed osteoderm based on histology from Hill (2006) and Krmpotic et al. (2015). (**E**) *Chaetophractus villosus* osteoderm and (**F**) histology based on Krmpotic et al. (2009). (**G**) Glyptodon sp. osteoderms (2) micro-CT of superficial and deep faces, showing similar shape as *D. novemcinctus*. (**H**) Paleohistology based on Wolf et al. (2012) of *Glyptodon clavipes. D asypus novemcinctus* specimen from the Burke Museum of Natural History and Culture (Seattle, WA, USA). *Glyptodon* sp. Specimen donated for scanning by T. Stankowich. Phylogeny based on Delsuc et al. (2016). (A) Attributions (top to bottom): *D. novemcinctus*: by lwolfartist, CC BY 2.0, via Wikimedia Commons; Glyptodon asper: by Arentderivative work: WolfmanSF, CC BY-SA 3.0, via Wikimedia Commons; Chaetophractus villosus: by Paul Prior, CC BY 4.0, via Wikimedia Commons; Chlamyphorus truncatus: by Daderot, CC0, via Wikimedia Commons; Priodontes maximus: by Colorado State University Libraries, CC BY-SA 4.0, via Wikimedia Commons; Tolypeutes tricinctus; by IQRemix from Canada, CC BY-SA 2.0, via Wikimedia Commons. (**B**) Attribution: *D. novemcinctus*: by MUSE, CC BY-SA 3.0, via Wikimedia Commons.

Our understanding of osteoderms primarily stems from studying reptiles (Moss 2008; Kirby et al. 2020; Williams et al. 2022; Yenmiş and Ayaz 2023; Daza et al. 2024; Maliuk et al. 2024; Ebel et al. 2025), but other groups, such as fishes and mammals, also have these structures (Vickaryous and Sire 2009; Yang et al. 2013). Armadillos (Order Cingulata), for example, stand out as the sole known extant lineage of mammals to possess a carapace of osteoderms, offering the opportunity to understand the evolution and function under vastly different physiological conditions relative to reptiles (Vickaryous and Sire 2009; Krmpotic et al. 2015). In armadillos, each osteoderm tile incorporates cavities that house hair follicles and sebaceous glands—features not found in reptilian dermal armor (Fig. 1; Vickaryous and Hall 2006; Krmpotic et al. 2015). While reptile osteoderms show broad variation in shape, size, and arrangement across taxa (Williams et al. 2022), armadillo osteoderms remain relatively conserved in both morphology and organization. This constancy may reflect the constraints of integrating rigid armor into a flexible, hair-bearing mammalian integument.

All members of the order Cingulata have an ossified carapace composed of osteoderms, but there is variation in both the overall organization of the carapace and the micro- and internal structure of individual osteoderms (Fig. 1; Krmpotic et al. 2015). Armadillos are divided into 2 families: Dasypodidae and Chlamyphoridae (Fig. 1A). In the Dasypodidae, the carapace is separated into 2 distinct regions defined by distinct osteoderm shapes; a fixed buckler region made up of hexagonal tiles located in the anterior (scapular) and posterior (pelvic) areas, and a series of movable imbricated bands in the abdominal midsection made up of overlapping rectangular tiles (Fig. 1B, D). Chlamyphoridae displays a greater diversity in osteoderm morphology and arrangement, with species-specific variations in the size, shape, of the osteoderms (Fig. 1E-F). The fixed buckler tiles in this family can be hexagonal, rectangular, or pentagonal, depending on the species. Some species have clear distinctions between fixed and movable regions (e.g., 3-banded armadillos), while other superficially appear more homogenous (e.g., giant and pink fairy armadillos; Fig. 1A). This family also includes extinct glyptodonts (subfamily †Glyptodontinae) which had enormous and ridged carapaces made up of hexagonal tiles (Fig. 1A, G; Delsuc et al. 2016). Across both families, osteoderms are held in place by Sharpey’s fibers (fibrillar collagen primarily composed of types I, III, and V; Fig. 1D) along their lateral edges that attach to adjacent osteoderms (Jones and Boyde 1974; Hill 2006; Wolf et al. 2012; Krmpotic et al. 2015). In addition, Dasypodidae has reduced bone projections at syndesmotic joints compared to Chlamyphoridae at these connection sites (Krmpotic et al. 2015).

Armadillo carapaces combine repeated, mineralized elements with compliant underlying attachments that create a tough yet flexible armor that shields against predators, cushions against burrow collapse, flexes for digging, and even allows one genus (Tolypeutes) to curl into a protective ball (McBee and Baker 1982; Vickaryous and Hall 2006; Chen et al. 2011; Ciancio et al. 2019; Marshall et al. 2021). Studies on glyptodonts (Alexander et al. 1999; du Plessis et al. 2018) and extant 9-banded armadillos (*D. novemcinctus* family Dasypodidae; Lee et al. 2010; Rhee et al. 2010; Chen et al. 2011) highlight how hierarchical layering, internal boney struts, and hard-soft tissue interfaces all modulate performance under tensile and/or compressive stress. Beyond mechanical protection against puncture and abrasion, armadillo osteoderms house sebaceous glands, piliferous hairs, yellow marrow, bone marrow, and nerve bundles which may aid thermoregulation by maximizing heat loss while minimizing heat production in hot environments (Fig. 1E, F; McNab 1980; Carlini et al. 2009; Superina and Loughry 2012; Feijó et al. 2020). This is supported by paleohistology that shows variation in these internal structures is associated with climatic-environmental conditions in different armadillo lineages (Fig. 1G, H; Ciancio et al. 2019). Dasypodidae diverged early within Cingulata and has reduced internal osteoderm cavity complexity but maintained a relatively similar external tile shape and carapace arrangement (Fig. 1D, F). The differences and similarities in Cingulata osteoderm anatomy lead us to ask: does the shape, connective tissue anatomy, and embedment of osteoderms in soft tissues collectively influence armor performance?

Using engineering techniques to evaluate the performance of armadillo armor is useful for modeling the contributions of the structural and material components of the carapace. For example, tensile and shear testing of hydrated and dried biological samples showed the material’s behavior under stress (Chen et al. 2011). Stability analysis on manufactured models mimicking armadillo armor showed tile size, underlying soft layer stiffness, and friction govern stability of individual tiles (Martini and Barthelat 2016a). Numerical modeling and drop weight impact tests on manufactured segmented models showed that impact energy is dissipated, and stability is increased when hard tiles have a soft substrate (Du et al. 2021). Systematic testing on cylindrical carapace models described how deformation directional patterns can be predicted through tile geometry, while soft joint connections increase stability in articulation (Ward Rashidi et al. 2019). Integrating biological principles, such as hierarchical layering, to these engineering methods enhances our understanding of how structural complexity supports functional performance. To gain a deeper understanding of how armor tissues synergize under different loading regimes, we must closely examine their anatomical details and develop models that capture their hierarchical complexity.

In this study, we quantify how tile shape, connective tissues, and soft dermis contribute to damage resistance and flexibility in the armadillo carapace using bio-inspired, multi-material models. Our goals were three-fold: (1) determine if armadillo osteoderm morphology serves a specific function by measuring the effect of different bioinspired tile shapes on bending stiffness, (2) quantify the effect of flexible vs. stiff connective fibers between tile segments, and (3) measure the role inter-tile material plays on the bending and puncture performance of the armor-fiber-soft tissue composite. We asked what happens when complexity is incrementally added: First, by incorporating connective fibers of varying tensile stiffnesses, and then by introducing a soft, dermal layer. This stepwise, bioinspired strategy not only deepens our understanding of natural armor but also offers a pathway for engineers to integrate these nuanced, multifunctional design elements into next-generation protective materials.

## Materials and methods

### Specimen and CT

Armadillo carapace specimens were obtained and micro-computed tomography (CT) scanned using a Bruker SkyScan 1173 micro-CT (μCT) machine (Karel F. Liem Bio Imaging Center at Friday Harbor Laboratories, WA, USA) (Table 1). Specimens were scanned with an Al 1.0 mm filter and a scanning resolution of 2k. Scans were reconstructed with Nrecon and visualized with 3D slicer (v5.6.2).

**Table 1:** Specimens micro-CT scanned

Species	Collection	Description	Scanner	Magnification (um)	Voltage (kV)	Current (uA)	Exposure (ms)
*D. novemcinctus*	Burke	Section of buckler osteoderm	Bruker 1173	25.9	60	133	1350
*D. novemcinctus*	SNOMNH	Section of buckler osteoderm	Bruker 1173	33	65	123	1150
*Glyptodon sp*.	T. Stankowich	Two fossilized osteoderms	Bruker 1173	23	129	61	1120
*G. floridanum*	T. Stankowich	Fossilized osteoderm	Bruker 1173	22	129	61	1120
*H. floridanus*	T. Stankowich	Fossilized osteoderm	Bruker 1173	28	129	61	1220

Sam Noble Oklahoma Museum of Natural History (SNOMNH), California State University, Fullerton (CSUF), Burke Museum of Natural History and Culture (Burke).

### Fabrication

**Fig. 2:**
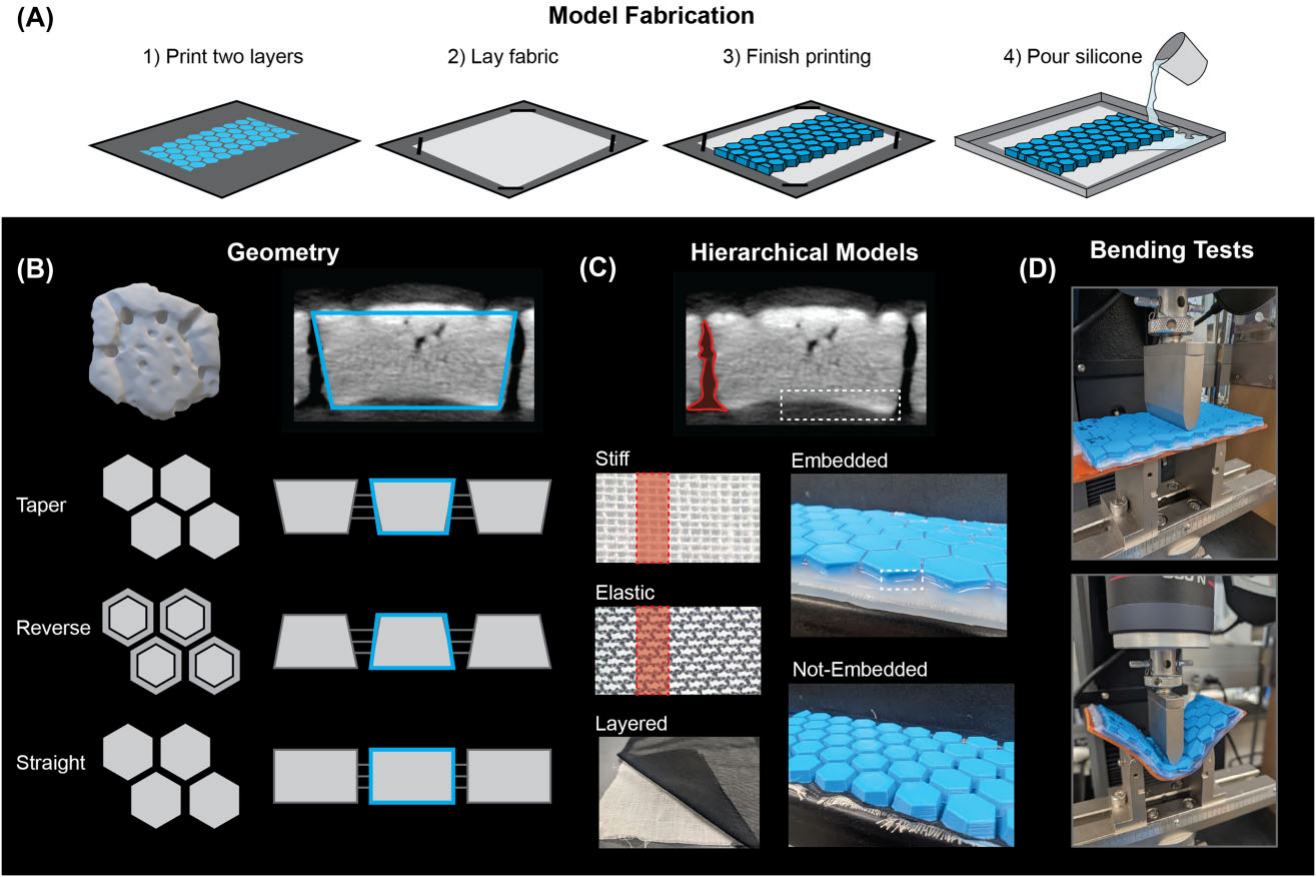
Hierarchical modeling and mechanical testing of armadillo-inspired segmented armor: (**A**) Overview of model fabrication. (**B**) Three variations of taper angles based on the geometry of *D. novemcinctus* buckler osteoderms. (**C**) Sharpey’s fibers and dermal connections were modeled using stiff, elastic, and layered fabrics. Models were then either embedded in silicon or left unembedded. Silicone embedded model photo is of the taper geometry and stiff fabric. Not-embedded model has reverse geometry and layered fabrics. (**D**) Models in testing position.

We created a set of bioinspired models using commercially available materials with different tile geometries, fabric types, and silicone (Fig Fig. 2A). By using known materials, we eliminated the unpredictable effects of viscoelastic biological materials, allowing us to focus solely on how shape affects mechanics.

Micro-CT scans showed that osteoderm tile morphology had an approximately 15% larger superficial face than deep face resulting in a tapered shape. To understand the mechanical effect this taper angle has on bending mechanics, our first tile design matched the generalized shape and angle found in the animal (Fig. 2B; “taper” model; top width 4.6 mm, bottom width 3.9 mm, height 2 mm). We tested 2 additional model shapes to assess whether the taper plays a role in bending performance. The second model had equivalent superficial and deep face widths (Fig. 2B; “straight” model uniform width 4.6 mm, height 2 mm). The third model was constructed with a reversed anatomy (narrower along the superficial face and wider along the deep face) compared to our initial tapered model (Fig. 2B; “reverse” model; top width 4 mm, bottom width 4.6 mm, height 2 mm). We designed each of our bio-inspired tiles in SolidWorks (v2025) as repeating arrays of 5 tiles by 10 tiles with 0.2 mm spacing between each individual tile. Models were printed using polylactic acid (PLA) on a Prusa Mk3s printer (Prusa Research, Prague, CZ) at 3× scale. We refer to these different shapes as “geometry” in our statistical analyses and plots.

To mimic connective tissues (e.g., collagen, ligaments) in armadillos, we used models as previously described. On these prints, we paused 3D printing after the first 2 PLA layers were printed and laid a piece of fabric onto the print bed prior to resuming the PLA printing (Fig. 2A, C). This results in the fabric becoming fixed into the printing material, allowing for a flexible and tessellated 3D print. We made 3 versions of these models, (1) an elastic fabric (Triple Textile, 80% Nylon/20% Spandex) mimicking the contribution of collagen fibers, (2) a stiff fabric (eFond cheesecloth, 100% Cotton) mimicking less stretchy connective tissues (ligaments), and (3) a layered fabric (a combination of both materials; elastic + stiff fabric model) to understand how rigid and elastic fibers might work together. For the layered fabric model, the stiff fabric was laid down following the first 2 PLA printed layers (as described previously), then the PLA printer printed one additional layer before the elastic fabric was added. We then resumed the rest of the PLA printing. For our statistics and plots, we refer to these different fabric arrangements as “fabric” treatments.

Finally, to simulate the soft tissues between and beneath osteoderms, we created models with 2 embed conditions: one with no simulated soft tissue, and one embedded in a soft platinum-catalyzed silicone rubber Ecoflex™-30 (Smooth-On Inc) to simulate soft tissue. Our 5 × 10 tile arrays were laid into a 13″ x 9″ baking tray that already had a layer of 160 g of silicone partially cured. We then mixed and poured another 160 g of silicone rubber into the tray so that it filled the spaces between the tiles and reached, but did not cover, the tops of the tiles. The models were weighed down by placing a second baking tray with similar dimensions on the superficial surface to ensure they were uniformly embedded into the silicone. All models were allowed to cure for at least 4 h (the pot life of Ecoflex™-30) before the trays were removed and testing could commence. For our statistics and plots, we refer to these different silicone conditions as “embed condition.”

We generated models with 3 separate geometries (straight, tapered, and reverse). Each of the 3 geometries were tested with 3 fabric variations (stiff, elastic, or layered; 9 total model combinations for geometry and fabric). These 9 models were tested with 2 embedding variations; embedded in silicone (4 replicates) or not-embedded (6 replicates) for a total of 18 separate models (Supplemental Table 1). The entire experiment consisted of 90 total tests.

### Material testing and analysis

Models were tested using an Instron Universal Testing Machine (5942 Single Column Universal Testing System, Norwood, MA, USA) in a three-point bending configuration (span = 60 mm; Fig 2D). Three-point bending allows us to quantify bending performance and evaluate how a material balances strain (to absorb energy) and stiffness (to resist loads). This trade-off is critical in both engineered armor (e.g., 3DDAI Kevlar composites optimized for delamination resistance and rigidity; Zheng et al. 2022) and natural armor systems (e.g., fish scales that allow for mobility without compromising protection; Yang et al. 2013; Martini et al. 2017). Models were tested on the interior curve to produce significant stiffening (Martini and Barthelat 2016b). Load (N), displacement (mm), and time (s) were measured and analyzed in R (v4.4.2)/R studio (v2024.12.0 + 467) using the CrshR github repository (https://github.com/CDonatelli/CrshR) and force–displacement curves were generated (Fig. 6 and Supplementary Fig. 1). From the data, the deformation of each model was calculated as flexural strain using


\begin{eqnarray*}
\varepsilon = \frac{{6\ d\ t}}{{{L^2}}},
\end{eqnarray*}

where *d* is the displacement (m), *t* is the thickness of the model (m), *L* is the span (m). To enable standardized comparisons between models, we calculated flexural strain at 7 N, the highest force common to all models (“flexural strain at 7 N”). To see the ultimate deformation each model underwent during testing, we also calculated the flexural strain at max load using the displacement at max load (“flexural strain at max load”). Each model’s resistance to bending was calculated as flexural stiffness (Nm2) using


\begin{eqnarray*}
E{\mathrm{I}} = \frac{{{{F\ }}{{{L}}^3}}}{{48\ {{d}}}},
\end{eqnarray*}

where *F* is the applied force (N), *L* is the span (m), and *d* is the displacement (m) at max load. The R-functions used to calculate the final values from the raw UTM data are available at: github.com/CDonatelli/Teeple-et.al.-2025.

### Statistics and Plotting

To determine the effects geometry, fabric, and silicone embed condition have on material properties, we ran separate three-way Analysis of Variance (ANOVA) in R for (1) flexural stiffness, (2) strain at max load, and (3) strain at 7 N (Wickham et al. 2023). We ran post-hoc pairwise tests using the pairwise.t.test function to determine which combinations of geometry, fabric, and silicone embed condition differed from each other (R stats, version 4.2.2) (RStudio: RStudio Team, 2020; R Core Team, 2021). Geometry pairs (tapered vs. straight, tapered vs. reverse, and reverse vs. straight) were tested separately for each fabric type (stiff, elastic, and layered) and silicone embed condition (embedded and non-embedded) yielding 18 total comparisons (e.g., tapered vs. straight under embedded stiff fabric models). Similarly, all fabric pairs (stiff vs. elastic, stiff vs. layered, and elastic vs. layered) were tested separately for each geometry (straight, tapered, and reverse), and silicone embed condition (embedded and non-embedded), yielding 18 additional tests (e.g, stiff vs. elastic for straight non-embedded models). In total, this approach resulted in 36 total comparisons for each bending parameter we looked at (Tables 3, 4 ,5). All pairwise *t*-tests were adjusted using the Bonferroni method.

Figures were generated with functions from the ggplot2, ggpubr, patchwork, and kableExtra packages in R (Kassambara 2023; Wickham et al. 2023; Pedersen 2024; Zhu et al. 2024).

## Results

### Overall comparisons

All 3 categories (Geometry, Fabric, and Silicone Embed Condition) had a significant effect on strain and flexural stiffness (Table 2). The two-way interactions between these categories were significant except for the interaction between Fabric x Embed Condition on Strain (Table 2). The three-way interaction was only significant for strain at 7 N (*P* = 0.04; Table 2). We explored the nature of these interactions with post hoc pairwise t-tests below.

**Table 2:** Three-way ANOVA table

		Strain at set load (7N)		Strain at max load		Flexural stiffness	
	DF	*F*	*P*-value	*F*	*P*-value	*F*	*P*-value
Fabric	2	41.477	**<0.001**	187.156	**<0.001**	815.590	**<0.001**
Geometry	2	65.067	**< 0.001**	333.636	**<0.001**	1050.977	**<0.001**
Embed	1	317.022	**<0.001**	101.874	**<0.001**	201.409	**<0.001**
Fabric x Geometry	4	5.227	**<0.001**	52.304	**<0.001**	104.630	**<0.001**
Fabric x Embed	2	19.994	**<0.001**	0.950	0.392	6.725	**0.002**
Geometry x Embed	2	22.450	**<0.001**	5.093	**0.009**	6.418	**0.003**
Fabric x Geometry x Embed	4	2.647	**0.040**	2.308	0.066	2.334	0.064
Residuals	72						

*F*-values for variables (fabric, geometry, and embedded silicone) and for their interaction effects (fabric x geometry, fabric x silicone, and geometry x silicone) for flexural strain at 7N, flexural strain at max load, and flexural stiffness.

### Flexural strain at 7N

**Fig. 3:**
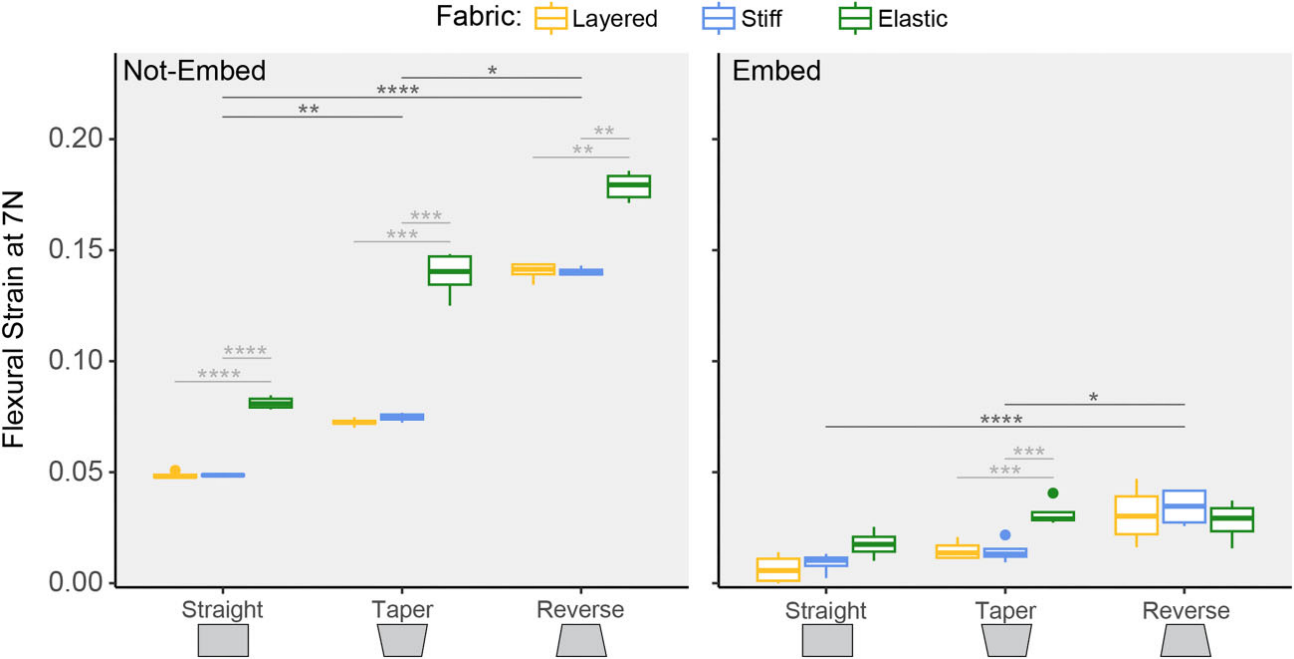
Flexural strain at a set load of 7 N. These plots show the averages and standard deviations for strain (unitless) required for models to reach a load of 7N in bending. The *x*-axis shows the different tile geometries and the different fabric conditions are denoted by color. Left is non-embedded models and right is silicone embedded models. Dark gray horizontal lines indicate significant differences between geometries of averaged fabric conditions. Light gray horizontal lines indicate significant differences between fabric conditions of each geometry. Significance codes: *P* < 0.05 “*,” *P* < 0.01 “**,” *P* < 0.001 “***,” *P* < 0.0001 “***.”

Stiff and layered fabrics had similar flexural strains at 7 N regardless of silicone embed condition or geometry (Fig. 3, Table 3). In non-embedded models, stiff and layered fabrics had lower strains at 7 N than elastic fabric across all geometry conditions (Fig. 3, Table 3). In silicone embedded models, elastic fabric was only different from stiff and layered fabrics in the taper geometry (Fig. 3, Table 3).

**Table 3:** Pairwise *t*-test *P*-values for flexural stain at 7 N

Comparison of geometry			Comparison of fabrics		
Non-embedded					
Layered	T vs. R	**<0.001**	Taper	Stf vs. Lyr	>0.999
	T vs. S	**<0.001**		Stf vs. Elas	**<0.001**
	R vs. S	**<0.001**		Elas vs. Lyr	**<0.001**
Stiff	T vs. R	**<0.001**	Straight	Stf vs. Lyr	>0.999
	T vs. S	**<0.001**		Stf vs. Elas	**<0.001**
	R vs. S	**<0.001**		Elas vs. Lyr	**<0.001**
Elastic	T vs. R	>0.999	Reverse	Stf vs. Lyr	>0.999
	T vs. S	**0.004**		Stf vs. Elas	**0.005**
	R vs. S	**0.002**		Elas vs. Lyr	**0.005**
Silicone embedded					
Layered	T vs. R	0.103	Taper	Stf vs. Lyr	>0.999
	T vs. S	0.662		Stf vs. Elas	**0.004**
	R vs. S	**0.012**		Elas vs. Lyr	**0.005**
Stiff	T vs. R	**0.005**	Straight	Stf vs. Lyr	>0.999
	T vs. S	0.826		Stf vs. Elas	0.23
	R vs. S	**0.001**		Elas vs. Lyr	0.08
Elastic	T vs. R	>0.999	Reverse	Stf vs. Lyr	>0.999
	T vs. S	0.082		Stf vs. Elas	>0.999
	R vs. S	0.248		Elas vs. Lyr	>0.999

Geometry comparisons for each fabric type (left) and fabric comparisons for each geometry type (right). Models not embedded into silicone rubber (above) and embedded into silicone (below).

**Fig. 4:**
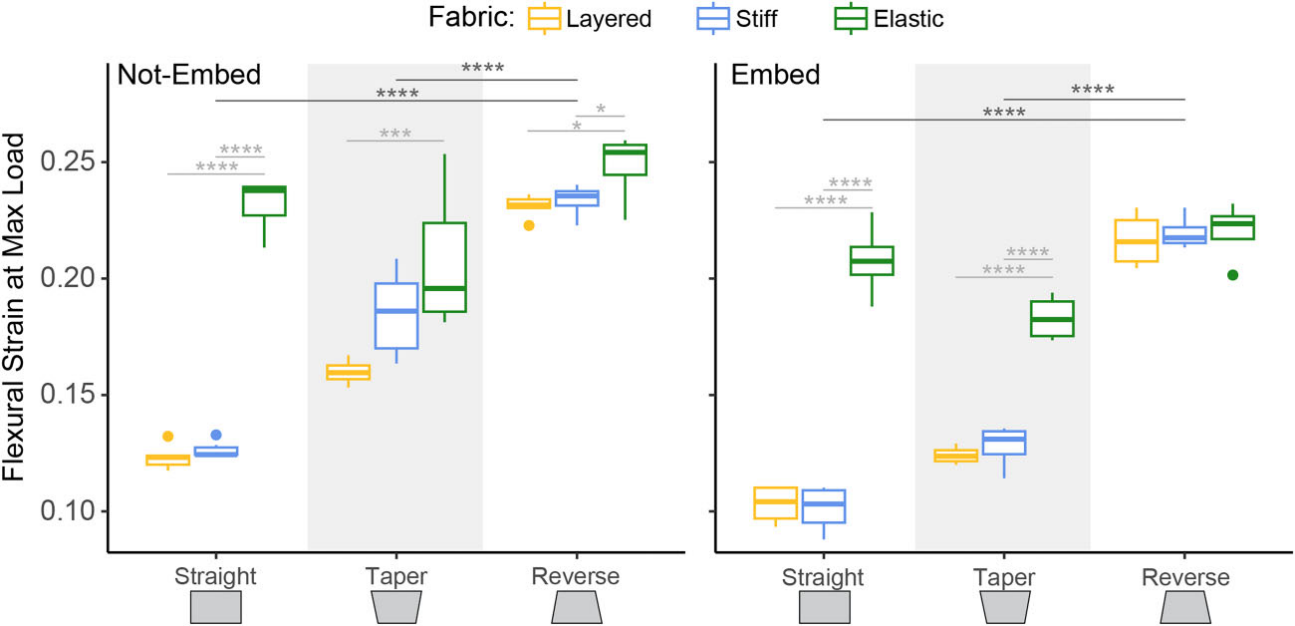
Flexural strain at max load. These plots show the averages and standard deviations for strain (unitless) at max load. The *x*-axis shows the different tile geometries and the different fabric conditions are denoted by color. Left is non-embedded models and right is silicone embedded models. Dark gray horizontal lines indicate significant differences between geometries of averaged fabric conditions. Light gray horizontal lines indicate significant differences between fabric conditions of each geometry. Vertical gray bars indicate significant differences between embed conditions. Significance codes: *P* < 0.05 “*,” *P* < 0.01 “**,” *P* < 0.001 “***,” *P* < 0.0001 “****.”

Reverse geometry had the highest flexural strains at 7N, while straight geometry tended to be the lowest (Fig. 3, Table 3). Non-embedded models had a more pronounced gap in strain between reverse and straight geometries (Fig. 4). Silicone embedded models showed that reverse remained the highest strain. Straight and taper geometries, however, were not significantly different from each other when embedded (Fig. 3, Table 3). All silicone embedded models had lower strains compared to non-embedded models (Fig. 3).

### Flexural strain at max load

Stiff and layered fabrics had similar flexural strain values at maximum load for each combination of silicone embed condition and geometry (Fig. 4, Table 4). Stiff and layered fabrics generally had lower strains than elastic fabrics except in 2 cases, (1) in non-embedded taper geometry, stiff and elastic fabrics did not differ and (2) in silicone embedded reverse geometry, stiff, elastic, and layered fabrics did not differ (Fig. 4, Table 4).

**Table 4:** Pairwise *t*-test *P*-values for flexural stain at max load

Comparison of geometry			Comparison of fabrics		
Non-embedded					
Layered	T vs. R	**<0.001**	Taper	Stf vs. Lyr	0.137
	T vs. S	**<0.001**		Stf vs. Elas	0.238
	R vs. S	**<0.001**		Elas vs. Lyr	**0.003**
Stiff	T vs. R	**<0.001**	Straight	Stf vs. Lyr	>0.999
	T vs. S	**<0.001**		Stf vs. Elas	**<0.001**
	R vs. S	**<0.001**		Elas vs. Lyr	**<0.001**
Elastic	T vs. R	**0.006**	Reverse	Stf vs. Lyr	>0.999
	T vs. S	0.126		Stf vs. Elas	**0.030**
	R vs. S	0.475		Elas vs. Lyr	**0.011**
Silicone embedded					
Layered	T vs. R	**<0.001**	Taper	Stf vs. Lyr	>0.999
	T vs. S	**0.026**		Stf vs. Elas	**<0.001**
	R vs. S	**<0.001**		Elas vs. Lyr	**<0.001**
Stiff	T vs. R	**<0.001**	Straight	Stf vs. Lyr	>0.999
	T vs. S	**0.008**		Stf vs. Elas	**<0.001**
	R vs. S	**<0.001**		Elas vs. Lyr	**<0.001**
Elastic	T vs. R	**0.011**	Reverse	Stf vs. Lyr	>0.999
	T vs. S	0.087		Stf vs. Elas	>0.999
	R vs. S	0.681		Elas vs. Lyr	>0.999

Geometry comparisons for each fabric type (left) and fabric comparisons for each geometry type (right). Models not embedded into silicone rubber (above) and embedded into silicone (below).

Reverse geometry had higher flexural strains than taper for each combination of fabric and silicone embed condition (Fig. 4, Table 4). Reverse geometry had higher flexural strains than straight in both layered and stiff fabrics (Fig. 4, Table 4). Taper had higher flexural strains than straight in layered and stiff fabrics (Fig. 4, Table 4). Non-embedded tapered models had a higher flexural strain than silicone embedded tapered models (Fig. 4).

### Flexural stiffness

**Fig. 5:**
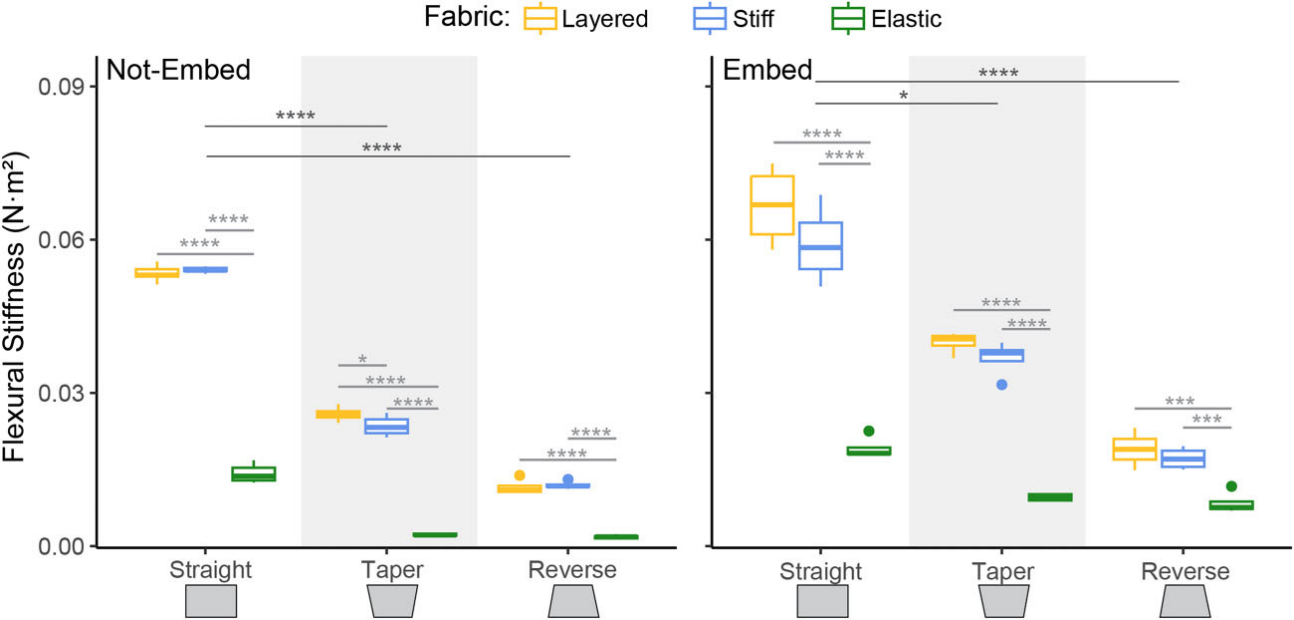
Flexural Stiffness (Nm2) at max load. These plots show the averages and standard deviations for flexural stiffness (Nm2) at max load. The *x*-axis shows the different tile geometries and the different fabric conditions are denoted by color. Left is unembedded models and right is silicone embedded models. Dark gray horizontal lines indicate significant differences between geometries of averaged fabric conditions. Light gray horizontal lines indicate significant differences between fabric conditions of each geometry. Vertical gray bars indicate significant differences between embed conditions. Significance codes: *P* < 0.05 “*,” *P* < 0.01 “**,” *P* < 0.001 “***,” *P* < 0.0001 “****.”

Stiff and layered fabrics had similar flexural stiffness across all geometries except in the non-embed taper condition, where layered fabric had slightly higher stiffness (Fig. 5 , Table 5). Layered and stiff fabrics were more resistant to bending than elastic fabric (Fig. 5, Table 5). Straight geometries consistently had higher stiffness than tapered and reverse geometries. Silicone embedded tapered models had higher stiffness than non-embedded tapered models; however, embedding did not affect stiffness in straight and reverse geometries.

**Fig. 6:**
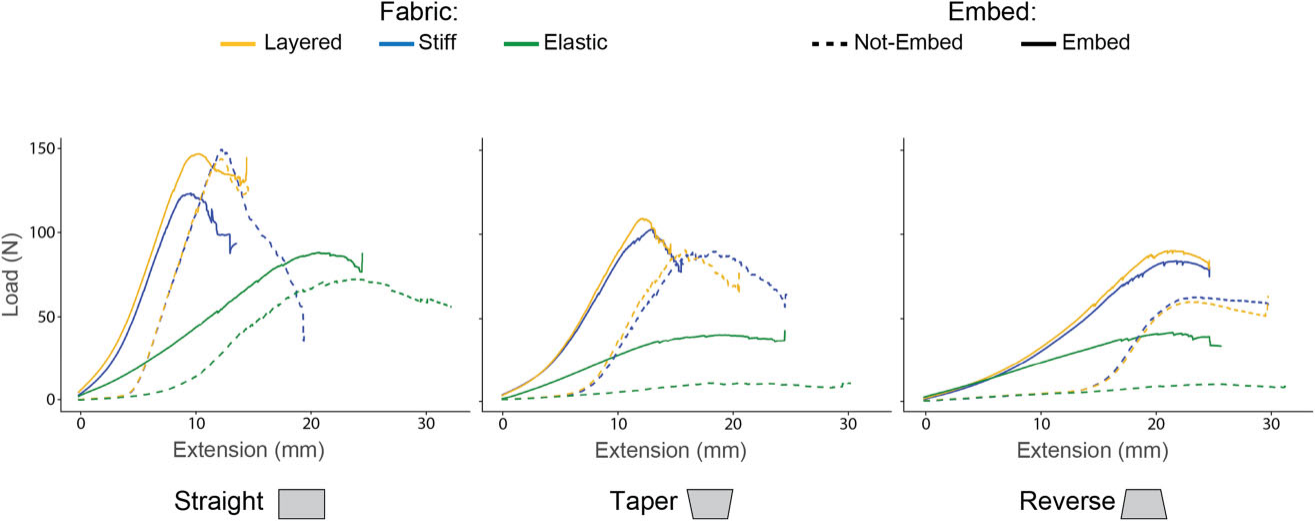
Average load extension curve for each model combination.

**Table 5:** Pairwise *t*-test *P*-values for flexural stiffness

Comparison of geometry			Comparison of fabrics		
Non-embedded					
Layered	T vs. R	**<0.001**	Taper	Stf vs. Lyr	**0.023**
	T vs. S	**<0.001**		Stf vs. Elas	**<0.001**
	R vs. S	**<0.001**		Elas vs. Lyr	**<0.001**
Stiff	T vs. R	**<0.001**	Straight	Stf vs. Lyr	>0.999
	T vs. S	**<0.001**		Stf vs. Elas	**<0.001**
	R vs. S	**<0.001**		Elas vs. Lyr	**<0.001**
Elastic	T vs. R	>0.999	Reverse	Stf vs. Lyr	>0.999
	T vs. S	**<0.001**		Stf vs. Elas	**<0.001**
	R vs. S	**<0.001**		Elas vs. Lyr	**<0.001**
Silicone embedded					
Layered	T vs. R	**<0.001**	Taper	Stf vs. Lyr	0.31
	T vs. S	**<0.001**		Stf vs. Elas	**<0.001**
	R vs. S	**<0.001**		Elas vs. Lyr	**<0.001**
Stiff	T vs. R	**0.001**	Straight	Stf vs. Lyr	0.41
	T vs. S	**<0.001**		Stf vs. Elas	**<0.001**
	R vs. S	**<0.001**		Elas vs. Lyr	**<0.001**
Elastic	T vs. R	>0.999	Reverse	Stf vs. Lyr	>0.999
	T vs. S	**<0.001**		Stf vs. Elas	**0.004**
	R vs. S	**<0.001**		Elas vs. Lyr	**0.001**

Geometry comparisons for each fabric type (left) and fabric comparisons for each geometry type (right). Models not embedded into silicone rubber (above) and embedded into silicone (below).

## Discussion

Armadillo armor is not a static shell; it is a dynamic system where rigid osteoderms and soft tissues work synergistically to respond to mechanical forces. Shape matters for armadillo armor—a shell made from tessellated, hexagonal tiles improves impact energy absorption, and an underlying soft interface is key for effectively distributing forces and resisting fracture damage (Martini and Barthelat 2016a; Du et al. 2021). Micro-CT imaging revealed that within the deep dermal layers, *D. novemcinctus*, armor has a reduced deep face size, resulting in a tapered tile morphology. Modeling this three-dimensional shape of the entire osteoderm (from the surface to the deepest layers), rather than simply focusing on the surface shape profile, is necessary to describe tile–tile and tile–soft tissue interactions. Mechanical testing of such multi-material models demonstrated that there are complex interactions at play.

Our data show that the natural tapered geometry similar to *D. novemcinctus* osteoderms consistently falls in the middle of the 2 other extremes (straight and reverse geometry; Figs. 3, 4, 5). This suggests that there is a “sweet spot” between being so stiff that you are unable to flex your armor without cracking and so flexible that external forces damage your internal tissues. Iteratively adding fabrics with variable degrees of compliance, representing the stiff ligamentous attachments and the stretchy collagen layers, demonstrated that the connections between tiles are critical for predicting bending resistance (Fig. 5). Adding in a pliable silicone layer representing the dermis reduces the degree of strain on the entire system and is likely critical for redistributing the small external forces that the animal experiences regularly (e.g., compression from sediment along the carapace while digging, puncture by prickly brambles when moving through vegetation). We found that the silicone layer reduced the effect of both fiber type and geometry when tested under these low force conditions (Fig. 3). The material properties of the integument between and beneath the carapace likely play a larger role in the way the armor system responds to large forces, like those imposed by a large predator, as these differences were not as pronounced at max load (Fig. 4).

Like our human engineered armors, natural armors must deal with the challenges of material limitations, durability concerns, impact distribution, and joint design. Often the same tile-tile interactions that increase flexibility result in decreased puncture resistance (Martini et al. 2017). The tapered morphology in *D. novemcinctus* armor allows an engineered material to bend more easily than one with straight-edged tiles; however, the smaller deep face of the tile might result in a less stable tile. Indeed, *D. novemcinctus* armor has been observed experiencing tilt failures where breaks follow the Sharpey’s fibers and entire tiles pop out along their perimeter (Lee et al. 2010). This bears the question: does the increased flexibility from the taper come with a trade-off to mechanical protection? Better understanding of *D. novencinctus* armor performance against laceration or puncture is needed to understand the balance between mobility and protection. Our bio-inspired models show that the surrounding tissues likely play a key role in armor performance. We suggest that future work should include investigations into the contributions of these tissues to armor performance and to test whether they serve to mitigate this mechanical tradeoff between flexibility and strength.

Armadillo species in closed habitats are exposed to predators with higher bite forces and develop thicker armor than species living in open habitat (Stapp 2022 M.S. thesis). While the elusive tendency of armadillos makes behavioral data limited (McDonough and Loughry 2013), it’s possible that variation in armor thickness may relate to antipredator strategies across species. The balling behavior commonly associated with armadillo is only present in one genus (3-banded armadillos: Tolypeutes spp.; Marshall et al. 2021). The rigid interlocking armor needed to ball up and withstand multiple predator bites seems to restrict maneuverability and speed in these species. Instead of balling, *D. novencinctus*, opts to sprint away from predators or, more dramatically, leap 4 feet into the air—a behavior observed in Lacandón Maya narrative and by Texans trying to avoid these animals on the road (Gilbert 1995; Newman and Rossi 2023). Given that this alternative strategy prioritizes maneuverability, selection may have favored thinner armor elements in *D. novencinctus* that prioritize mobility to enable quick escape behaviors. Different genera of armadillo from both families should be tested in a similar hierarchical way to understand the relationship between 3-dimensional armor geometry and defensive strategy.

*Dasypus novemcinctus* armor has orientation-specific characteristics that balance flexibility and structural integrity across the different loading regimes they face. External forces pressing inward on armor, such as those from impact, need to be absorbed without excessive deformation. While forces coming from the inside out, for example, forces generated internally by the animal’s own musculoskeletal system, require reduced bending resistance to enable motion. Testing models with the taper geometry represents the performance of *D. novemcinctus* armor against external forces; while testing models with reverse geometry tiles simulates the internal forces that act on the armor. *Dasypus novemcinctus* asymmetric tile shape can provide a balance in performance where greater flexibility is allowed from internal forces, while rigidity is maintained for external forces, ensuring that the armor remains protective without catastrophically reducing mobility.

To the best of our knowledge, no prior studies have explicitly investigated taper morphology in other armadillo species. While our observations confirmed a tapered morphology in extant Dasypodidae, it appears to be absent in extant Euphractinae (Family Chlamyphoridae; Krmpotic et al. 2009, 2015; Ciancio et al. 2019) Fossil records of fixed buckler osteoderms reveal substantial variation in osteoderm tapering, suggesting that a broad taxonomic sampling with multiple specimens is needed to investigate its ancestral condition. For example, within Glyptodonts and Pampatheres (Family Chlamyphoridae), *Glyptotherium floridanum* (Hill 2006) and *Glyptodon clavipes* (Wolf et al. 2012) seem to have a taper while *Glyptodon reticulatus* (Hill 2006) and *Propalaehoplophorus sp*.(Wolf et al. 2012) do not. It appears taper and straight are the predominate variations throughout Cingulata; however, we did find one instance of what seems to be reversed taper morphology (superficial face smaller than the deep face) in *Sadypus sp*. (Family Chlamyphoridae; Ciancio et al. 2019). Taper morphology also occurs in other taxa. Some lizard osteoderms exhibit a taper morphology (larger superficial surface relative to their deep face) similar to what we observed in *D. novemcinctus* (Willan 2024). Given the differences in integumentary physiologies between mammals and reptiles, this convergent morphology may reflect a mechanical adaptation rather than a physiological constraint. However, to investigate the evolutionary origins, a combination of mechanical testing, which is limited in reptile osteoderms (Williams et al. 2022), and phylogenetic mapping across Cingulata and squamates would be needed.

The ability of armored structures to flex without failing should depend on both how the tiles are connected to each other and how they connect to the animal. Our models serve as first order approximations for determining the relationships between the geometry of hard tiles, connective fabrics (connective tissues), and pliable silicone embedded layers (soft tissues). We aim to use the general principles learned from this study to inform future bio-inspired models and to predict the relationships between hard and soft tissues in armadillos. The way osteoderm tiles lay in the skin varies by animal. Armadillos have a tight arrangement of tiles that form a distinct shell, while many reptile species have osteoderms that move more freely with the skin. We found that our models’ stiff connective fibers set a hard limit on bending, regardless of whether they are paired with more elastic materials. There is no additive effect—once these rigid fibers reach their threshold, further flexibility is restricted. However, when only highly elastic connections are present, we observed a sliding effect at the osteon level, allowing for greater mobility between tiles.

This work highlights 2 essential ideas in bio-inspired design: (1) model design matters, and (2) the importance of interdisciplinary collaboration. Our models dissect the hierarchical nature of biological structures into a framework for engineering design. It was crucial when designing our models to pick shapes, materials, and arrangements that answered the questions we wanted to ask. For example, if we ignored the soft tissues between tiles by omitting our silicone embed condition, we would not have learned that the soft tissues between tiles dampen forces (Fig. 3), work in conjunction with the taper of the tiles to increase resistance to bending (Fig. 5) and reduce deformation under high loads (Fig. 4). If we had simply used our most elastic fabric type, we would not have learned that it is the combination of different connective tissue types that holds tiles in place (Fig. 4) and stiffens the armor-skin composite.

These findings would not have been possible without the combination of techniques and questions designed by both Biology and Engineering. Engineering techniques such as model design and manufacturing and material testing informed us about how materials affect mechanics. But without the use of techniques like CT scanning, dissection, and histology, we would have no biological basis to design these models, resulting in data with no biological relevance. It is important to consider techniques from across disciplines when outlining experiments to answer these types of questions. Only then will we be able to truly understand the intricate designs found in nature.

## Supplementary Material

icaf055_Supplemental_Files

## Data Availability

julia.teeple@csu.fullerton.edu and cassandra.donatelli@gmail.com https://github.com/CDonatelli/Teeple-et.al.-2025

## Notes

From the symposium “Evolution to innovation: Bridging biology and engineering through bioinspired design” presented at the annual meeting of the Society for Integrative and Comparative Biology, January 3–7, 2025.
