# Supplementary material for: Tapered Tiles Modulate Flexibility in Segmented Armadillo-Inspired Armor
Source: Integr Comp Biol. 2025 May 27;65(6):1562–75. doi: 10.1093/icb/icaf055 (PMC12690472; doi:10.1093/icb/icaf055)
Supplement: icaf055_Supplemental_Files [file icaf055_supplemental_files.zip › icb-2025-0059-File014.docx]

**Supplementary Table 1.** Samples used for testing with average and standard deviation of model weight.

| Geometry | Fabric | Embed condition | Weight (g) | Number of replicates |
| --- | --- | --- | --- | --- |
| Taper | Stiff | Embedded | 61.71 ± 5.83 | 4 |
|  | Stiff | Non-embedded | 27.01 ± 0.06 | 6 |
|  | Stretch | Embedded | 56.97 ± 4.97 | 4 |
|  | Stretch | Non-embedded | 26.73 ± 0.05 | 6 |
|  | Combination | Embedded | 65.21 ± 4.70 | 4 |
|  | Combination | Non-embedded | 27.89 ± 0.17 | 6 |
| Straight | Stiff | Embedded | 58.39 ± 4.93 | 4 |
|  | Stiff | Non-embedded | 30.45 ± 0.20 | 6 |
|  | Stretch | Embedded | 60.14 ± 3.82 | 4 |
|  | Stretch | Non-embedded | 29.62 ± 0.48 | 6 |
|  | Combination | Embedded | 62.14 ± 6.43 | 4 |
|  | Combination | Non-embedded | 31.58 ± 0.12 | 6 |
| Reverse Taper | Stiff | Embedded | 53.98 ± 6.22 | 4 |
|  | Stiff | Non-embedded | 26.50 ± 0.11 | 6 |
|  | Stretch | Embedded | 60.47 ± 2.57 | 4 |
|  | Stretch | Non-embedded | 29.62 ± 0.48 | 6 |
|  | Combination | Embedded | 56.18 ± 6.08 | 4 |
|  | Combination | Non-embedded | 27.49 ± 0.21 | 6 |
