# Supplementary figures and images for: Tapered Tiles Modulate Flexibility in Segmented Armadillo-Inspired Armor
Source: Integr Comp Biol. 2025 May 27;65(6):1562–75. doi: 10.1093/icb/icaf055 (PMC12690472; doi:10.1093/icb/icaf055)

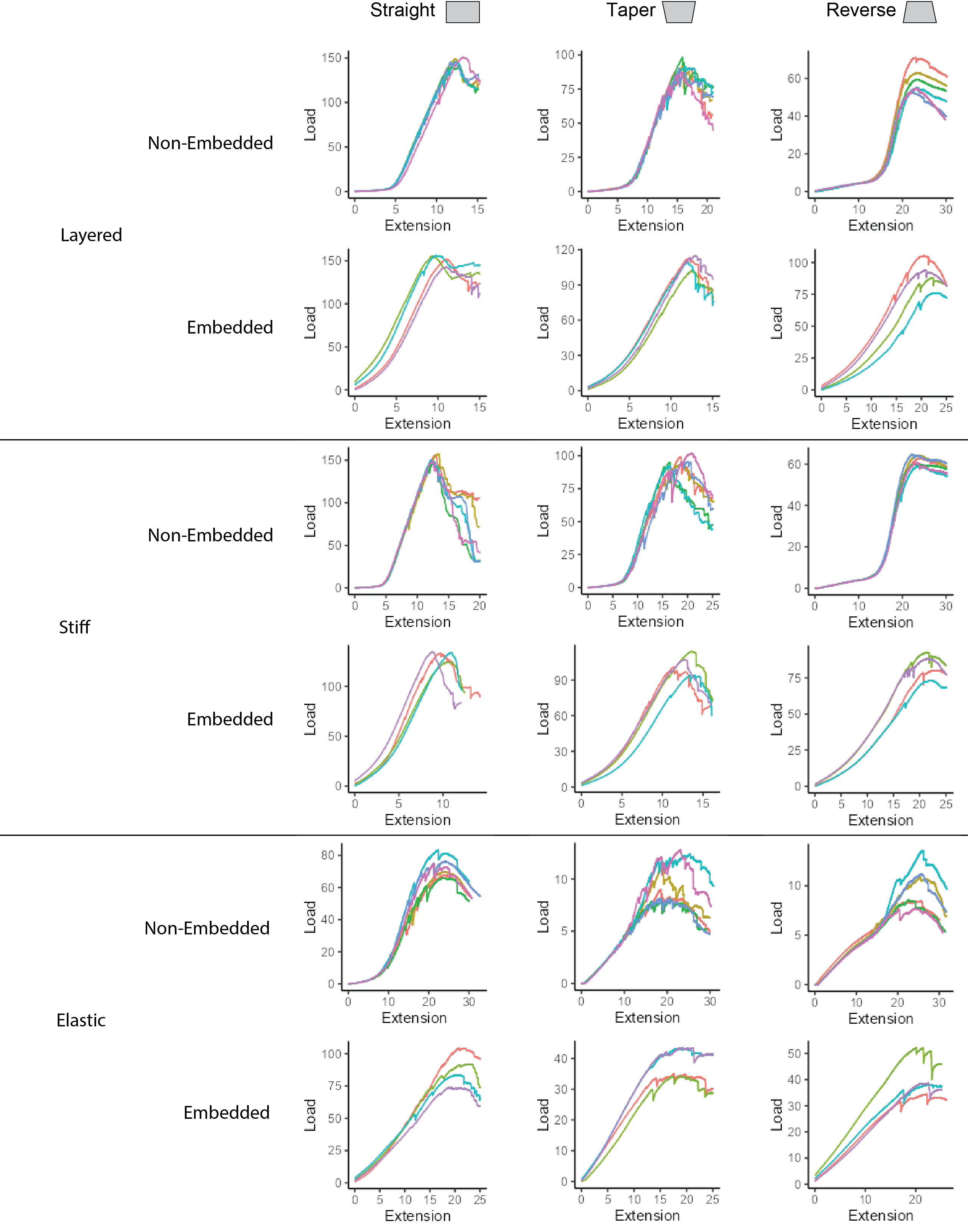

Supplement: icaf055_Supplemental_Files [file icaf055_supplemental_files.zip › icb-2025-0059-File013.png]
